# Understanding Oceanic Migrations with Intrinsic Biogeochemical Markers

**DOI:** 10.1371/journal.pone.0006236

**Published:** 2009-07-22

**Authors:** Raül Ramos, Jacob González-Solís, John P. Croxall, Daniel Oro, Xavier Ruiz

**Affiliations:** 1 Departament Biologia Animal (Vertebrats), Universitat de Barcelona, Barcelona, Spain; 2 British Antarctic Survey, Natural Environment Research Council, Cambridge, United Kingdom; 3 Instituto Mediterráneo de Estudios Avanzados (IMEDEA), CSIC-UIB, Mallorca, Spain; NOAA/NMFS/SWFSC, United States of America

## Abstract

Migratory marine vertebrates move annually across remote oceanic water masses crossing international borders. Many anthropogenic threats such as overfishing, bycatch, pollution or global warming put millions of marine migrants at risk especially during their long-distance movements. Therefore, precise knowledge about these migratory movements to understand where and when these animals are more exposed to human impacts is vital for addressing marine conservation issues. Because electronic tracking devices suffer from several constraints, mainly logistical and financial, there is emerging interest in finding appropriate intrinsic markers, such as the chemical composition of inert tissues, to study long-distance migrations and identify wintering sites. Here, using tracked pelagic seabirds and some of their own feathers which were known to be grown at different places and times within the annual cycle, we proved the value of biogeochemical analyses of inert tissue as tracers of marine movements and habitat use. Analyses of feathers grown in summer showed that both stable isotope signatures and element concentrations can signal the origin of breeding birds feeding in distinct water masses. However, only stable isotopes signalled water masses used during winter because elements mainly accumulated during the long breeding period are incorporated into feathers grown in both summer and winter. Our findings shed new light on the simple and effective assignment of marine organisms to distinct oceanic areas, providing new opportunities to study unknown migration patterns of secretive species, including in relation to human-induced mortality on specific populations in the marine environment.

## Introduction

Understanding spatiotemporal dynamics of marine vertebrates is essential to determine when and where animals are exposed to human impacts [1], [2]. We have now clear signs that human activities and resulting global changes are having a strong impact on marine ecosystems [3]. Contamination episodes and massive fishery activities, such as oil spills or longlining, are responsible for the direct death of hundreds of thousands of marine vertebrates worldwide, leading to overall population declines of many shark, turtle, dolphin, seal and seabird species [4], [5]. Global warming is also inducing changes in the distribution and abundance of marine prey and will therefore affect the distribution and movements of their predators [6], [7]. Assessing the spatial interaction between these threats and marine predators will therefore be critical for effective conservation management. In migratory predators, this means not only assessing their distribution and abundance over time, but also their movements between breeding, feeding and wintering areas.

Although recent advances in tracking technology are helping to fill the current gap in marine migration knowledge, studies are usually restricted to a few individuals often tracked for short periods due to logistical and financial constraints [8]–[13]. As a result, there is increasing interest in using intrinsic markers to identify and link breeding and wintering sites of a large variety of marine predators [10], [14], [15]. In this regard, biogeochemical intrinsic markers, such as stable isotope signatures or element concentrations, can be particularly useful for studying migration dynamics, as no other intrinsic marker (i.e. biometrics or genetics) can identify wintering areas [16].

Migrating birds with known moulting patterns provide a singular opportunity to validate the utility of biogeochemical markers in the marine environment. As feathers grow, the elements and their isotopic forms assimilated through the diet are incorporated into the keratin structure. Once formed, feathers become metabolically inert, thus integrating the composition of the local food web where feathers were grown [17]. Many studies using biogeochemical markers have recently attempted to link wintering and breeding populations of different bird species along terrestrial environments [18], [19]. However, hindered by the difficulty of determining wintering grounds in the open ocean, seabirds and ocean isotopic landscapes have attracted less attention [20], [21]. Nevertheless we can now track seabirds over the entire annual cycle using geolocators, which allows us to relate breeding and wintering areas to the geochemical composition of specific feathers.

Twice a year, millions of seabirds travel tens of thousands of kilometres across the equator to move between wintering and breeding areas, enhancing their susceptibility to threats posed by human activities [2]. As a result, pelagic seabirds are becoming increasingly threatened at a faster rate globally than all other species-groups of birds [5].

**Figure 1:**
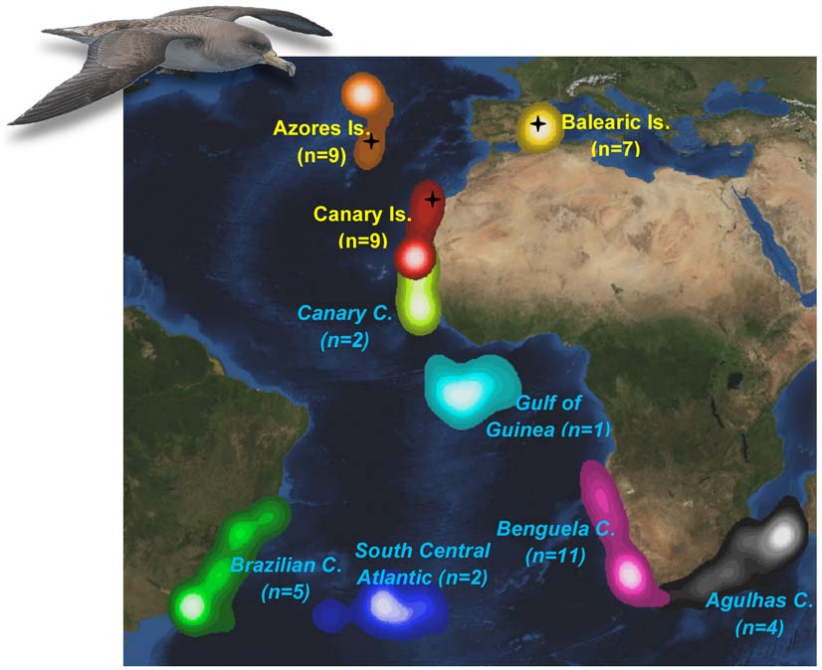
Studied breeding and wintering sites of Cory's shearwater. Main foraging areas of Cory's shearwaters at the end of the breeding season, between August and October (legends in yellow), the period when most Cory's shearwaters grow the first primary feather and during the wintering season, between December and January (legends in light blue), when most shearwaters grow the eighth secondary feather [22], [23]. Activity ranges are derived from kernel analyses encompassing from 5 (light tone) to 90% (dark tone) of validated locations. Number of birds included in each area is shown in brackets. Sampling sites are shown with black crosses. Picture courtesy of Albert Cama.

Here, we explored the value of using biogeochemical analyses as intrinsic markers to understand long distance movements of vertebrates in the marine environment. To do so, we studied Cory's shearwaters *Calonectris diomedea*, a long-distance migrant that breeds on temperate northeast Atlantic and Mediterranean Islands and winters in major upwelling areas of the Atlantic Ocean [13] (Fig. 1). To elucidate the integration of isotopes and elements from different food webs into their tissues, we first tracked 25 shearwaters over the entire annual cycle using light level geolocators, allowing us to identify both the breeding and the wintering areas for each bird. Second, we determined the stable isotope signatures of carbon (δ^13^C), nitrogen (δ^15^N), sulphur (δ^34^S), hydrogen (δ^2^H ) and oxygen (δ^18^O) and the elemental concentrations of selenium (Se), lead (Pb) and mercury (Hg) in feathers from the tracked birds moulted at the end of the breeding season and at the wintering grounds [22], [23]. Using both sets of information, we demonstrate that isotopic signatures and element composition in feathers reflect the signatures of water masses where they were grown.

## Results and Discussion

We found that breeding birds mostly foraged within a few hundreds of kilometres of their colony sites (mean distance to the colony in August: Azores Is.: 380.1±96.7 km; Balearic Is.: 297.0±86.8 km; Canary Is.: 442.7±121.4 km; Fig. 1). Selected populations for this study are separated by several thousand kilometres and located in different oceanographic regimes [24]. In this regard, primary feathers grown at the end of the breeding period differed in their composition among breeding sites in stable isotope signatures (ANOVA: δ^13^ C, *F_2,14_* = 66.6, *P*<0.001; δ^15^ N, *F_2,12_* = 40.1, *P*<0.001; δ^34^ S, *F_2,15_* = 146.8, *P*<0.001; δ^2^ H, *F_2,14_* = 74.0, *P*<0.001; δ^18^ O, *F_2,12_* = 48.5, *P*<0.001) as well as in elementary content (ANOVA: Se, *F_2,14_* = 11.8, *P* = 0.001; Pb, *F_2,12_* = 7.3, *P* = 0.009; Hg, *F_2,12_* = 12.6, *P* = 0.001). These differences allowed us to assign any individual to a relatively restricted breeding area using isotopic signature (100% correct classification) and, to a lesser extent, elemental composition (72.0% correct classification) of primary feathers (Table 1). Although the studied populations do not include the entire known breeding distribution for the species [mainly missing the central and eastern Mediterranean; 25], with the results presented here we can assign with confidence the geographic origin (at large scale) of these shearwaters using the biogeochemical values of their breeding feathers.

**Table 1:** Discriminant classification based on feather biogeochemistry.

	Stable isotopes P1	Stable isotopes S8	Element analysis P1	Element analysis S8
**Breeding colonies**				
*Original data*				
Azores Is. (n = 9)	100.0	44.4	66.7	66.7
Balearic Is. (n = 7)	100.0	57.1	71.4	100.0
Canary Is. (n = 9)	100.0	66.7	88.9	88.9
Total (n = 25)	100.0	56.0	76.0	84.0
*Cross-validation*				
Azores Is. (n = 9)	100.0	22.2	66.7	55.6
Balearic Is. (n = 7)	100.0	28.6	57.1	85.7
Canary Is. (n = 9)	100.0	55.6	88.9	55.6
Total (n = 25)	100.0	36.0	72.0	64.0
**Wintering sites**				
*Original data*				
Benguela C. (n = 11)	63.6	90.9	45.5	36.4
Brazil-Falklands C. (n = 5)	80.0	100.0	60.0	40.0
Agulhas C. (n = 4)	75.0	100.0	75.0	50.0
Canary C. (n = 2)	100.0	100.0	100.0	100.0
SC Atlantic (n = 2)	100.0	100.0	50.0	100.0
Total (n = 24)	75.0	95.8	58.3	50.0
*Cross-validation*				
Benguela C. (n = 11)	54.5	63.6	27.3	27.3
Brazil-Falklands C. (n = 5)	20.0	60.0	40.0	0.0
Agulhas C. (n = 4)	25.0	100.0	25.0	50.0
Canary C. (n = 2)	100.0	100.0	100.0	100.0
SC Atlantic (n = 2)	100.0	0.0	0.0	50.0
Total (n = 24)	50.0	66.7	33.4	33.4

Correct classification rates (%) obtained using stable isotope analysis (δ^13^C, δ^15^N, δ^34^S, δ^2^H and δ^18^O) and element concentrations (Se, Pb and Hg) on summer (P1) and winter (S8) feathers. Discriminant analyses were cross validated using jackknife procedures. The Gulf of Guinea wintering area was not included in this analysis because it was visited by only a single bird.

**Figure 2:**
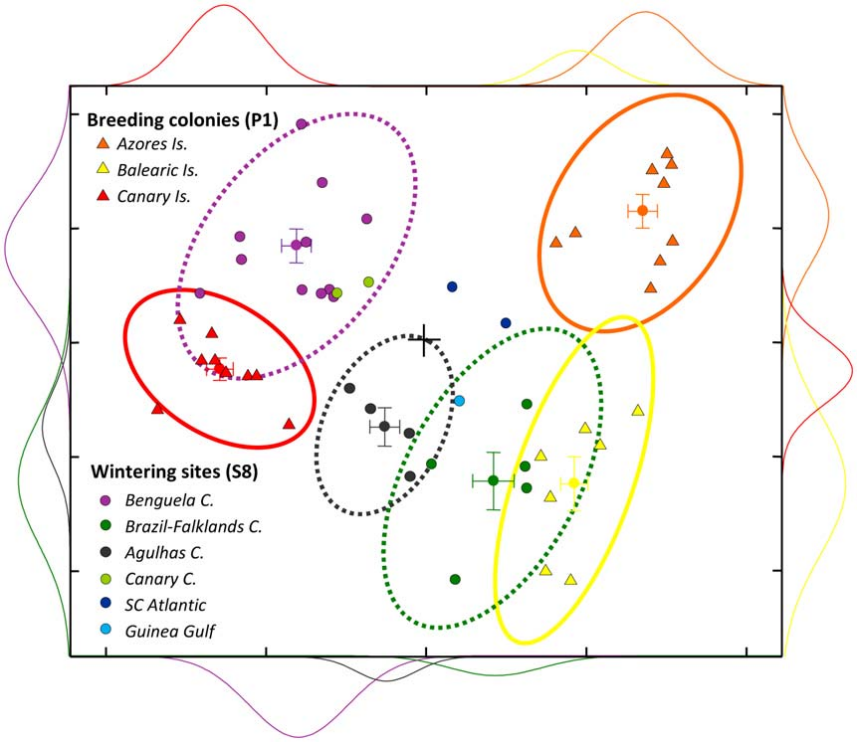
Isotopic composition of summer and winter feathers. Principal Component Analysis (PCA) of stable isotopic signatures of carbon (δ^13^C), nitrogen (δ^15^N), sulphur (δ^34^S), hydrogen (δ^2^H ) and oxygen (δ^18^O) in first primary (P1) and eighth secondary (S8) feathers (triangles and circles, respectively) of Cory's shearwaters moulted in breeding and wintering areas respectively. X-axis represents PC1 (59.0%) while Y-axis represents PC2 (21.1%); both are unitary divided with zeros on the middle cross-intersection. Gaussian bivariate ellipses (95% probability interval of the mean population) and normal distribution curves are shown.

In winter, birds travelled to the central and south Atlantic, concentrating in one of the six wintering areas (Fig. 1) associated with the Benguela (*n = *11), Brazil-Falklands (*n = *5), Agulhas (*n = *4), Canary (*n = *2) Currents, with the South Central Atlantic Ocean (*n = *2) and with the Gulf of Guinea (*n = *1). These oceanic areas were not different from those previously reported for the species [13], [26]–[28]. Since each area has its own distinctive oceanographic features [24], the isotopic signatures of the secondary feathers grown during the wintering period also differed among the main wintering areas (ANOVA: δ^13^ C, *F_2,7_* = 28.1, *P*<0.001; δ^15^ N, *F_2,9_* = 45.9, *P*<0.001; δ^34^ S, *F_2,7_* = 34.0, *P*<0.001; δ^2^ H, *F_2,8_* = 21.3, *P* = 0.001; δ^18^ O, *F_2,9_* = 2.36, *P* = 0.079) and could also be used to assign birds to specific wintering oceanic areas (66.7% correct classification; Table 1 and Fig. 2). In contrast, elemental analyses did not differ among wintering sites and showed a low rate of correct assignment (33.4%; ANOVA: Se, *F_2,7_* = 0.7, *P* = 0.54; Pb, *F_2,9_* = 1.5, *P* = 0.27; Hg, *F_2,6_* = 1.1, *P* = 0.38). Indeed, elemental analyses of feathers moulted in wintering areas were more successful in assigning birds to their breeding origin than to their wintering areas (64.0% vs. 33.4% of correct classification; Table 1). In fact, element concentrations of primary and secondary feathers are rather similar when grouped according to the breeding areas (Table S1). Such results confirm a differential behaviour in the accumulation and excretion dynamics between isotopic ratios and element concentrations [16], [21]. Whereas stable isotope signatures of feathers reflect an exogenous origin, *i.e.* they are promptly transferred from the diet to feathers when moulting [29], elemental burdens of feathers may indicate an endogenous origin of elements, *i.e.* they are partially mobilized from various organs where they are stored [30], [31]. Consequently, the interpretation of elemetal concentrations of migratory species from tissues formed out of the breeding season should be made with caution because those values could reflect exposures to elements during the breeding season, and vice versa. The deposition of elements acquired at breeding grounds into tissues grown out of the breeding period may be particularly important in species with long breeding seasons and relatively short wintering periods. In our case, Cory's shearwaters spend on average 243 days at the breeding grounds, but only 80 days on the wintering grounds [13].

Geographical variation in composition of tissues grown in distinct oceanic water masses can arise from different sources. Migrating predators may change diet between seasons, resulting in differences in trophic level and in isotopic values and element levels incorporated into the tissues [32]. Seasonal differences in foraging behaviour, such as inshore vs. offshore foraging, could also contribute to these differences [14], [33]. In some cases, natural biogeochemical gradients have also been described in the marine environment [34], [35]. Finally, as many biogeochemicals biomagnify throughout food chains, differential food web complexity among oceanographic systems has been identified as a prime source of geographical variation [36], [37], contributing to the characterization of specific oceanic water masses.

In summary, this study showed that by choosing the appropriate tissue, isotope ratios and element composition can be used to assign marine predators to specific oceanic regions used during the breeding or wintering periods. Feathers of long-distance migratory seabirds are often replaced in a predictable manner in different oceanic regions throughout their annual journeys [22], [38], [39], thus providing excellent opportunities to study their migration through biogeochemical analyses [e.g. 40]. For other non-avian migratory species, specific portions of tissues, such as hair, whiskers, nails, scales [41]–[44], sampled at a particular time within their annual cycle could also be used to provide biogeochemical information about breeding and wintering areas; however appropriate validations should be conducted. In organisms with long erythropoiesis and vitellogenesis processes (e.g. reptiles), even corpuscular blood (red blood cells) and yolk eggs can be used for such purposes [45], [46]. However, it is also evident that elemental concentrations acquired in one season could be transferred to tissues grown in another season, highlighting the need to consider the carry-over effect of elemental concentrations between distinct oceanic areas. Such results open new insights into migration routes of marine vertebrates and provide an effective tool that can be used to assign marine organisms to specific breeding and wintering areas. This information provides new opportunities to study human-induced mortality caused by activities, such as fisheries, oil spills or climatic changes, on specific populations.

## Methods

### Ethics Statement

All animals were handled in strict accordance with good animal practice as defined by the current European legislation, and all animal work was approved by the respective regional committees for scientific capture (Consejería de Medio Ambiente del Cabildo de Gran Canaria, Canary Is., Spain; Secretaria Regional do Ambiente da Região Autónoma dos Açores, Azores Is., Portugal; and Govern Balear, Balearic Is., Spain).

### Study design

Transoceanic migrations can currently be investigated using global location sensing (GLS) devices based on recording light levels, which can be deployed on a bird all year-round and will give 2 positions per day with an accuracy of 186±114 km [47]. This method can provide year round information on the location of breeding and wintering sites. In June and July 2002 we deployed 50 geolocators on Cory's shearwaters breeding in three geographically distant areas: Vila Islet (Azores Is.), Pantaleu Islet (Balearic Is., Mediterranean) and Veneguera (Gran Canaria, Canary Is.). After approximately one year we retrieved the GLS loggers and sampled the 1^st^ primary and 8^th^ secondary feathers from those birds that returned, obtaining year-round GLS data and feather samples from 9, 7 and 9 birds, respectively. Feathers were analysed for stable isotopes of carbon, nitrogen, sulphur, hydrogen and oxygen and for elemental concentrations of selenium, lead and mercury.

### Sample preparation and laboratory analyses

All feathers were washed in a 0.25 M sodium hydroxide solution, rinsed thoroughly in distilled water to remove any surface contamination, dried in an oven at 60°C to constant mass, and ground to a fine powder in a freezer mill (Spex Certiprep 6750; Spex Inc., Metuchen, New Jersey, USA) operating at liquid nitrogen temperature. Subsamples of 0.4 mg of feather powder for carbon and nitrogen, about 3.5 mg for sulphur, and 0.25 mg for hydrogen and oxygen analyses were weighed to the nearest µg, placed into tin and silver capsules and crimped for combustion. Samples were oxidized in a Flash EA1112 and TC/EA coupled to a stable isotope mass spectrometer Delta C and Delta Plus XL, respectively through a Conflo III interface (ThermoFinnigan, Bremen, Germany), where the δ^13^C, δ^15^N, δ^34^S, δ^2^H and δ^18^O values were determined. Isotope ratios are expressed conventionally as δ values in parts per thousand (‰) according to the following equation: δ*X* = [(*R*_sample_/*R*_standard_) - 1]×1000, where *X* (‰) is ^13^C, ^15^N, ^34^S ^2^H or ^18^O and *R* are the corresponding ratio ^13^C/^12^C, ^15^N/^14^N, ^34^S/^32^S, ^2^H/^1^H or ^18^O/^16^O related to the standard values. *R*_standard_ for ^13^C is Pee Dee Belemnite (PDB), for ^15^N is atmospheric nitrogen (AIR), for ^34^S is troilite of the Canyon Diablo Meteorite (CDT) and for ^2^H and ^18^O is Vienna Standard Mean Ocean Water (V-SMOW). The isotopic ratio mass spectrometry facility at the Serveis Científico-Tècnics of Universitat de Barcelona (Spain) applies international standards (IAEA CH_7_, IAEA CH_6_ and USGS 24 for C, IAEA N1, IAEA N2 and IAEA NO_3_ for N and IAEA-S1, IAEA-S2 and IAEA-S3 for S) while Duke Environmental Stable Isotope Laboratory of Duke University (USA) uses internal keratin standards previously calibrated against NIST and IAEA reference materials (CFS, BWB and CHS for H and O; Wassenaar and Hobson 2003), all of them inserted every 12 samples to calibrate the system and compensate for any drift over time. Replicate assays of standard materials indicated measurement errors of±0.1, ±0.2, ±0.3, ±1.5 and ±0.1‰ for carbon, nitrogen, sulphur, hydrogen and oxygen respectively but these are likely underestimates of true measurement error for complex organics like feathers.

To determine trace element concentrations, 50 mg of feather powder was digested in 1 ml of nitric acid (69–70%) and 0.5 ml of hydrogen peroxide (30%) using Teflon® bombs during 12 hours at 60°C. The result of the digestion was diluted into 7 ml of distilled water. Quantitative analysis was performed using the ICP-AES technique (atomic emission spectrometer, Perkin Elmer Optima 3200 RL, Connecticut, USA) at Serveis Científico-Tècnics of Universitat de Barcelona (Spain). Accuracy of analysis was checked by measuring certified reference material (Human Hair CRM 397, Community Bureau of Reference, Commission of the European Community).

### Statistical analyses

Element concentrations were log-transformed to achieve normality. Differences among breeding and among wintering populations in stable isotope and element values were tested with one-way ANOVA. Tests among wintering populations do not include the Canary Current, South Central Atlantic and Gulf of Guinea because fewer than four birds were found in each area. To assess whether feather composition could be linked to specific oceanic areas, we used classificatory discriminant analyses (SPSS 2003) on the composition of both type of feathers in relation the breeding and wintering areas. Discriminant analyses were carried out separately for stable isotope signatures of C, N, S, H and O and for combined element concentrations of Se, Pb and Hg. We tested models by jackknife cross-validation. Models were built step by step including independent variables according to the Wilks' Lambda criterion, and breeding and wintering areas were weighted according to the sample size.

## Supporting Information

Table S1 Biogeochemical composition of summer and winter feathers. Stable isotope signatures (‰) and log-transformed element concentrations (ng g-1) for primary feathers (P1) according to the breeding areas and for secondary feathers (S8) according to the wintering areas. Values are means±standard deviation and sample size is shown in brackets. Significant differences among breeding and among wintering populations are indicated by *** P<0.0001, ** P<0.05 and * P<0.1. ANOVA-test among wintering populations do not include Canary Current, South Central Atlantic and Gulf of Guinea. Standard coefficients from discriminant functions on original data (explained variance in brackets) are conducted separately for stable isotopes and element concentrations.
(0.06 MB DOC)
