# Supplementary material for: Understanding Oceanic Migrations with Intrinsic Biogeochemical Markers
Source: PLoS One. 2009 Jul 22;4(7):e6236. doi: 10.1371/journal.pone.0006236 (PMC2705790; doi:10.1371/journal.pone.0006236)
Supplement: Table S1 — Biogeochemical composition of summer and winter feathers. Stable isotope signatures (‰) and log-transformed element concentrations (ng g-1) for primary feathers (P1) according to the breeding areas and for secondary feathers (S8) according to the wintering areas. Values are means±standard deviation and sample size is shown in brackets. Significant differences among breeding and among wintering populations are indicated by *** P<0.0001, ** P<0.05 and * P<0.1. ANOVA-test among wintering populations do not include Canary Current, South Central Atlantic and Gulf of Guinea. Standard coefficients from discriminant functions on original data (explained variance in brackets) are conducted separately for stable isotopes and element concentrations. (0.06 MB DOC) [file pone.0006236.s001.doc]

[**Supplementary Table 1**](http://www.nature.com/nature/journal/v451/n7182/suppinfo/nature06518.html)**. Biogeochemical composition of summer and winter feathers.** Stable isotope signatures (‰) and log-transformed element concentrations (ng g^-1^) for primary feathers (P1) according to the breeding areas and for secondary feathers (S8) according to the wintering areas. Values are means ± standard deviation and sample size is shown in brackets. Significant differences among breeding and among wintering populations are indicated by ^***^ *P* < 0.0001, ^**^ *P* < 0.05 and ^*^ *P* < 0.1. ANOVA-test among wintering populations do not include Canary Current, South Central Atlantic and Gulf of Guinea. Standard coefficients from discriminant functions on original data (explained variance in brackets) are conducted separately for stable isotopes and element concentrations.

|  | **δ^13^C** | **δ ^15^N** | **δ ^34^S** | **δ ^2^H** | **δ ^18^O** | **Se** | **Pb** | **Hg** |
| --- | --- | --- | --- | --- | --- | --- | --- | --- |
| **Primary feathers (P1)** |  |  |  |  |  |  |  |  |
| Azores Is. (*n=*9) | -16.25±0.25 | 12.74±0.91 | 19.85±0.37 | 67.45±10.75 | 46.13±3.30 | 3.88±0.10 | 2.42±0.63 | 3.84±0.10 |
| Balearic Is. (*n=*7) | -16.19±0.17 | 10.19±0.67 | 19.55±0.21 | 40.00±9.94 | 30.01±7.52 | 3.88±0.10 | 2.87±0.44 | 4.08±0.21 |
| Canary Is. (*n=*9) | -13.69±0.62 | 12.77±0.39 | 17.22±0.37 | -7.59±14.56 | 25.62±5.61 | 3.65±0.12 | 2.15±0.24 | 3.64±0.13 |
| Total (*n=*25) | -15.31±1.31^***^ | 12.04±1.35^***^ | 18.82±1.27^***^ | 32.75±34.80^***^ | 34.23±10.70^***^ | 3.80±0.15^***^ | 2.45±0.53^**^ | 3.83±0.23^***^ |
| *Standardized Coefficients* |  |  |  |  |  |  |  |  |
| Discriminant function (85.4%) | -0.59 | -0.26 | 0.55 | 0.36 | 0.17 |  |  |  |
| Discriminant function (81.7%) |  |  |  |  |  | 0.25 | 0.48 | 0.76 |
| **Secondary feathers (S8)** |  |  |  |  |  |  |  |  |
| Benguela C. (*n=*11) | -14.64±0.51 | 14.47±0.50 | 17.56±0.66 | 27.19±14.96 | 25.13±5.21 | 3.77±0.11 | 1.78±1.03 | 3.83±0.19 |
| Brazil-Falklands C. (*n=*5) | -16.12±0.40 | 11.45±0.60 | 19.42±0.19 | 28.21±30.01 | 21.76±1.27 | 3.81±0.07 | 1.77±0.70 | 3.80±0.25 |
| Agulhas C. (*n=*4) | -15.85±0.11 | 13.16±0.54 | 19.00±0.61 | -2.68±2.20 | 22.08±1.87 | 3.71±0.17 | 2.43±0.58 | 3.62±0.24 |
| Canary C. (*n=*2) | -14.69±0.36 | 14.34±0.05 | 18.82±0.14 | 32.05±5.68 | 21.76±0.83 | 3.75±0.03 | 2.26±0.30 | 4.11±0.08 |
| SC Atlantic (*n=*2) | -15.53±0.60 | 12.81±0.47 | 18.89±0.07 | 45.39±0.12 | 31.55±1.45 | 3.66±0.04 | 2.33±0.01 | 3.73±0.05 |
| Gulf of Guinea (*n=*1) | -16.04 | 12.90 | 19.36 | 27.19 | 22.15 | 3.87 | 2.41 | 3.90 |
| Total (*n=*25) | -15.26±0.78^***^ | 13.45±1.28^***^ | 18.44±0.95^***^ | 24.46±20.40^***^ | 24.09±4.44^*^ | 3.76±0.11 | 1.99±0.81 | 3.81±0.22 |
| *Standardized Coefficients* |  |  |  |  |  |  |  |  |
| Discriminant function (86.4%) | 0.73 | 0.94 | -0.17 | -0.14 | -0.44 |  |  |  |
| Discriminant function (63.6%) |  |  |  |  |  | -0.36 | -0.67 | -0.36 |
